# Non-destructive identification of *Pseudostellaria heterophylla* from different geographical origins by Vis/NIR and SWIR hyperspectral imaging techniques

**DOI:** 10.3389/fpls.2023.1342970

**Published:** 2024-01-15

**Authors:** Tingting Zhang, Long Lu, Yihu Song, Minyu Yang, Jing Li, Jiduan Yuan, Yuquan Lin, Xingren Shi, Mingjie Li, Xiaotan Yuan, Zhongyi Zhang, Rensen Zeng, Yuanyuan Song, Li Gu

**Affiliations:** ^1^ Key Laboratory of Ministry of Education for Genetics, Breeding and Multiple Utilization of Crops, College of Agriculture, Fujian Agriculture and Forestry University, Fuzhou, China; ^2^ Key Laboratory of Biological Breeding for Fujian and Taiwan Crops, Ministry of Agriculture and Rural Affairs, Fujian Agriculture and Forestry University, Fuzhou, China; ^3^ Pharmaceutical Development Board of Zherong County, Ningde, China; ^4^ Huzhou Wuxing Jinnong Ecological Agriculture Development Co., Ltd, Huzhou, China

**Keywords:** *Pseudostellaria heterophylla*, geographical origin, hyperspectral imaging, machine learning, deep learning

## Abstract

The composition of *Pseudostellaria heterophylla* (Tai-Zi-Shen, TZS) is greatly influenced by the growing area of the plants, making it significant to distinguish the origins of TZS. However, traditional methods for TZS origin identification are time-consuming, laborious, and destructive. To address this, two or three TZS accessions were selected from four different regions of China, with each of these resources including distinct quality grades of TZS samples. The visible near-infrared (Vis/NIR) and short-wave infrared (SWIR) hyperspectral information from these samples were then collected. Fast and high-precision methods to identify the origins of TZS were developed by combining various preprocessing algorithms, feature band extraction algorithms (CARS and SPA), traditional two-stage machine learning classifiers (PLS-DA, SVM, and RF), and an end-to-end deep learning classifier (DCNN). Specifically, SWIR hyperspectral information outperformed Vis/NIR hyperspectral information in detecting geographic origins of TZS. The SPA algorithm proved particularly effective in extracting SWIR information that was highly correlated with the origins of TZS. The corresponding FD-SPA-SVM model reduced the number of bands by 77.2% and improved the model accuracy from 97.6% to 98.1% compared to the full-band FD-SVM model. Overall, two sets of fast and high-precision models, SWIR-FD-SPA-SVM and SWIR-FD-DCNN, were established, achieving accuracies of 98.1% and 98.7% respectively. This work provides a potentially efficient alternative for rapidly detecting the origins of TZS during actual production.

## 1 Introduction

*Pseudostellaria heterophylla* (Miq.) Pax, also known as Tai-zi-shen (TZS), is a perennial herbaceous plant belonging to the *Caryophyllaceae* family (Li et al., 2016). Its roots have a long history of use as medicinal and edible plants in Asian countries, including China and Korea. This plant is renowned for its safety and its content of polysaccharides, saponins, cyclopeptides, sterols, oils, and other volatile oily substances, which contribute to enhancing the human immune system (Wong et al., 1994). TZS is commonly employed as a substitute for ginseng and American ginseng, addressing issues such as loss of appetite and serving as a potent tonic. Wild TZS resources are widely distributed in various provinces of China, such as Fujian, Guizhou, Jiangsu, and Anhui. However, the composition of TZS varies among different geographical origins. To evaluate TZS quality based on polysaccharide and saponin content, it is crucial to consider cultivated plants from specific regions, such as Jiangsu Province and Fujian Province (Shi et al., 2013). Therefore, distinguishing the origins of TZS is significant.

Most traditional methods used to identify the origins and grades of herbs rely on external characteristics such as shape, color, microstructure, and odor. However, the similarity of the external features of TZS makes it difficult to detect their origins and grades, especially after processing (Wu et al., 2018). Currently, the identification of TZS is conducted through techniques like High-Performance Liquid Chromatography, Gas Chromatography-Mass Spectrometry, ninhydrin color, and other analytical methods that aim to detect specific active components (Lin et al., 2011). However, these methods are time-consuming, labor-intensive, expensive, and require the use of large quantities of organic solvents, which can potentially harm the environment. Thus, there is an urgent need for a rapid and accurate analytical method to determine the origins of TZS.

Today, hyperspectral imaging (HSI) is widely utilized in agri-food product quality and safety control (Lu et al., 2020; Tian et al., 2023). The HSI combines conventional imaging and spectroscopic techniques to present a hypercube with one spectral dimension and two spatial dimensions. This allows it to provide both spatial and spectral information about the sample (Zareef et al., 2021). This information is closely related to the chemical composition and physical properties of the sample (Delwiche and Kim, 2000). Therefore, the HSI technique has attracted considerable attention in distinguishing between similar groups of biological materials such as maize (Wang et al., 2022), wheat (Zhang et al., 2018; Zhang et al., 2022b), wolfberries (Zhang et al., 2020a; Dong et al., 2022).

Artificial intelligence technology has assumed a crucial role in numerous global domains. Machine learning (ML) is an essential approach in studying artificial intelligence. In recent years, the ML field has experienced a significant transformation owing to the development of novel deep learning (DL) classifiers. DL, with its capacity to comprehend intricate and representative patterns from vast datasets, has found applications across diverse domains. Shallow Convolutional Neural Networks (CNN), representative algorithms for DL, have been proven by previous studies to be ideal for analyzing and processing HSI data (Liu et al., 2020; Zhang et al., 2022a). The complexity of traditional neural networks is reduced by a simple network structure. The “end-to-end” design concept, coupled with the hidden-neuron network structure, empowers us to autonomously extract relevant data features and optimize large datasets for accurate target classification (Fu et al., 2018). To the best of the researcher’s knowledge, the combination of HSI and DL algorithms to recognize the geographical origins and quality grades of TZS has not been reported yet.

Therefore, in this study, we utilized HIS combined with DL and ML techniques for the evaluation of the geographical origins of *Pseudostellaria heterophylla* (Miq.) Pax (TZS). The successive projection algorithm (SPA) and competitive adaptive weighted sampling (CARS) were employed to extract spectral information that is highly correlated with the origins of TZS. ML methods, such as partial least squares discriminant analysis (PLS-DA), random forests (RF), and support vector machines (SVMs), were also compared as modeling approaches alongside deep convolutional neural network (DCNN) architectures.

## 2 Materials and methods

### 2.1 Sample preparation

**Figure 1:**
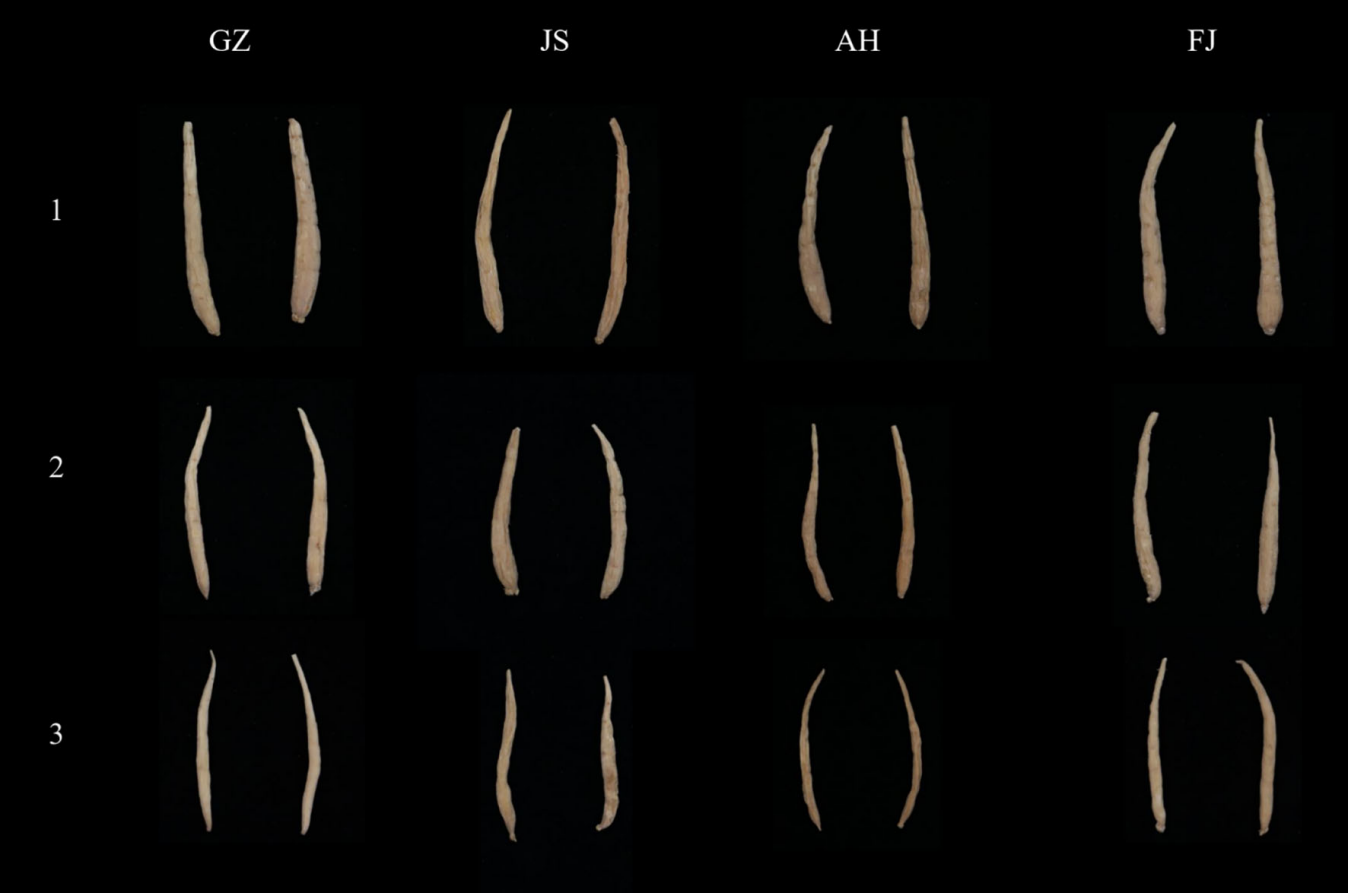
Typical TZS samples from Guizhou (GZ), Jiangsu (JS), Anhui (AH) and Fujian (FJ) Provinces. The numbers “1, 2, 3” on the left represent the different quality levels of TZS.

The TZS samples utilized in this study were collected from four distinct geographical regions in China, including Guizhou, Fujian, Jiangsu, and Anhui Provinces. To improve the applicability of the model, we attempted to increase the complexity of the sample composition. Two or three germplasm resources for each geographical region were selected for this reason, encompassing different quality grades of TZS (**Table 1**). We randomly selected 3249 samples from the TZS accessions, covering all three quality grades. The quality grades of TZS samples were determined according to the commercial grades for Chinese materia medica-PSEUDOSTELLARIAE RADIX (T/CACM 1021.127-2018). Specifically, the first-grade samples were characterized by roughly straight shapes, with diameters of the thickened root section greater than or equal to 0.4 cm and individual weights greater than or equal to 0.4 g. Similarly, the second-grade samples also had roughly straight shapes, with diameters of the thickened root section greater than or equal to 0.3 cm and individual weights greater than or equal to 0.2 g. In contrast, the third-grade samples were classified as bent, with diameters of the thickened root section below 0.3 cm and individual weights below 0.2 g. Additionally, to capture comprehensive spectral information of the TZS, both sides of each sample were scanned using visible and near-infrared (Vis/NIR) as well as shortwave infrared (SWIR) hyperspectral instruments. Consequently, a total of 12996 hyperspectral images of the TZS samples were obtained. Typical TZS images from different origins and quality grades are shown in **Figure 1**.

**Table 1:** The geographical origins and quality grades of TZS.

Geographical origins	Name	Year	Number of samples in different quality grades		
			1	2	3
Fujian province	Zheshen 1	2022	96	82	80
	Zheshen 4	2022	82	84	84
	Landrace 1	2023	104	104	102
Guizhou province	Guishen 1	2023	92	92	92
	Landrace 1	2023	96	88	90
	Landrace 2	2023	81	90	92
Jiangsu province	Landrace 1	2023	132	132	132
	Landrace 2	2023	132	136	134
Anhui province	Xuanshen 1	2023	92	90	92
	Xuanshen 2	2023	92	90	90
	Xuanshen 3	2023	92	90	92

### 2.2 Hyperspectral image acquisition and correction

The hyperspectral imaging (HSI) system comprised instruments for both Vis/NIR and SWIR spectral ranges. The Vis/NIR instrument consists of a GaiaField Pro-V10E spectrometer (Specim, Spectral Imaging Ltd., Finland), a high-resolution camera (Sichuan Dualix Spectral Imaging Technology Co., Ltd., China), and two 150 W halogen light sources. The SWIR instrument is composed of a GaiaField Pro-N17E-HR spectrometer (Specim, Spectral Imaging Ltd., Finland), a shortwave infrared high-resolution camera (Sichuan Dualix Spectral Imaging Technology Co., Ltd., China), and two 150 W tungsten halogen lamps. These two instruments employed a sample stage that was electrically positioned and controlled by a stepper motor. A computer equipped with SpecView Software (Sichuan Dualix Spectral Imaging Technology Co., Ltd., China) was provided. The instruments mentioned above were enclosed within a box featuring a black inner surface, thus constituting the HSI system.

To eliminate errors like baseline drift, the HSI system should be preheated by powering it on for 30 minutes before image collection. Subsequently, non-deformed three-dimensional hyperspectral images (x, y, λ), commonly known as “hypercubes”, were obtained by placing the TZS samples on the platform. To obtain high-quality hyperspectral reflectance images of the TZS samples, the Vis/NIR-HSI image acquisition parameters of motor speed, exposure time, and object distance were set at 1.18 cm/s, 7.2 ms, and 25 cm through several attempts. Similarly, the SWIR-HSI image acquisition parameters of motor speed, exposure time, and object distance were adjusted to 1.5 cm/s, 4.5 ms, and 25 cm after several attempts. Hyperspectral image correction was then conducted by using white and black references according to the method depicted in Zhang et al. (2020b).

### 2.3 Spectral data extraction

Each TZS sample was then considered as a region of interest (ROI) and was segmented from the black background by threshold segmentation. The difference between the sample and the background reflectance was maximum at the 801.05 nm band (Vis/NIR) and 1005.67 nm band (SWIR), so every sample was segmented at these bands separately. The spectra of pixels belonging to the same TZS sample were averaged to derive average spectra, which were then utilized for discrimination analysis purposes. The head and tail bands were eliminated from the spectra to minimize the effects of instability stemming from random noise. Consequently, 673 bands from 400.20 nm to 999.75 nm for the Vis/NIR and 482 bands from 900.96 nm to 1700.43 nm for the SWIR hypercubes were utilized for future analysis.

### 2.4 Spectral data preprocessing

To minimize the potential effects of overlapping or light noise across different spectral wavelengths (Alchanatis et al., 2005), as well as to assess the impact of various pre-processing methods on the classification of TZS origins, several spectral pre-processing techniques were investigated and applied to the raw spectra. The evaluated pre-processing techniques included standard normal variate (SNV) (Barnes et al., 1989), Detrend (DT), and Savitzky–Golay first derivative (FD) (Zhang et al., 2020b).

### 2.5 Multivariate data analysis

In this study, various machine learning algorithms were employed, including traditional two-stage methods such as partial least squares discriminant analysis (PLS-DA), support vector machine (SVM), and random forest (RF), as well as an end-to-end deep learning algorithm known as the deep convolutional neural network (DCNN). The purpose of these algorithms was to distinguish TZS samples into different origin groups.

#### 2.5.1 Traditional two-stage machine learning algorithms

PLS-DA is a widely practiced classifier that is considered a supervised technique that maximizes the distinction between different groups of samples (Nie et al., 2019). RF is an ensemble learning algorithm developed by Leo Breiman, inspired by the earlier work of Amit and Geman (Breiman, 2001). RF offers several advantages, including fast training speed, few tuning parameters, and the ability to handle high-dimensional data effectively. At its core, RF is a collection of decision trees that work together to make predictions (Tian et al., 2021). SVM is a non-probabilistic, linear, binary classifier used to classify linear and nonlinear data by learning a hyperplane. In nonlinear classification, SVM uses a kernel function to map original data to high-dimensional data and build hyperplanes to optimally classify the closest training samples in different classes (Burges and discovery, 1998; Wang et al., 2023). In this study, the radial basis function (RBF) kernel was selected for the SVM algorithm and the penalty coefficient *c* and kernel parameter *g* were optimized by a grid search procedure in the range of 2^−8^–2^8^ through five-fold cross-validation.

#### 2.5.2 Deep learning algorithms

The DCNN is a widely recognized deep learning architecture that is inspired by the visual perception mechanisms found in living organisms (Zhang et al., 2019). A one-dimensional DCNN was developed as the primary classifier to process the data from each source. The DCNN architecture consisted of three convolutional blocks, one flattening layer, and five fully-connected layers. Each convolutional block comprised convolutional, batch normalization, maximum pooling, and dropout layers. To extract local features from the spectral information effectively, while reducing its dimensions and enhancing non-linearity, we utilized 1×3 convolution kernels with a stride and padding of 1 (Yu et al., 2021). The first and second convolutional layers were configured with 32, 64, and 128 filters, respectively.

Utilizing rectified linear units (ReLUs) in the DCNN resulted in faster training and helped mitigate model overfitting compared to networks using older units (Nie et al., 2019). To facilitate learning and reduce the emphasis on initialization, batch normalization was applied before each dense layer and after each convolutional layer (Ioffe and Szegedy, 2015). The fully connected (Fc) layers were composed of 512, 128, 64, 32, and 4 neurons, respectively. To convert the DCNN output into probabilities for each category, a softmax function was introduced to the activation function of Fc5 (Kumar et al., 2022). The categorical cross-entropy loss function was employed to measure the distance between the probability distribution of the DCNN output values and the true values. To minimize the loss function, we utilized an adaptive moment estimation algorithm with a learning rate of 0.001, beta_1 of 0.9, and beta_2 of 0.99 (Yu et al., 2021).

### 2.6 Model implementation and evaluation

The sample data were randomly divided into training and validation sets in a ratio of 7: 3. The classification accuracy, which was used to evaluate the performance of the models, was determined by calculating the ratio of correctly classified samples to the total number of samples. The diagrams were developed using Origin Pro 9.0 (Origin Lab Corporation, Northampton, MA, USA). MATLAB R2019b (The MathWorks, Natick, MA, USA) was utilized for spectrum extraction, spectrum preprocessing, and ML model development. The DCNN model was built using Keras, a renowned deep-learning framework (https://keras.io/zh/).

## 3 Results and discussions

### 3.1 Reflectance spectral characteristics of the samples

**Figure 2:**
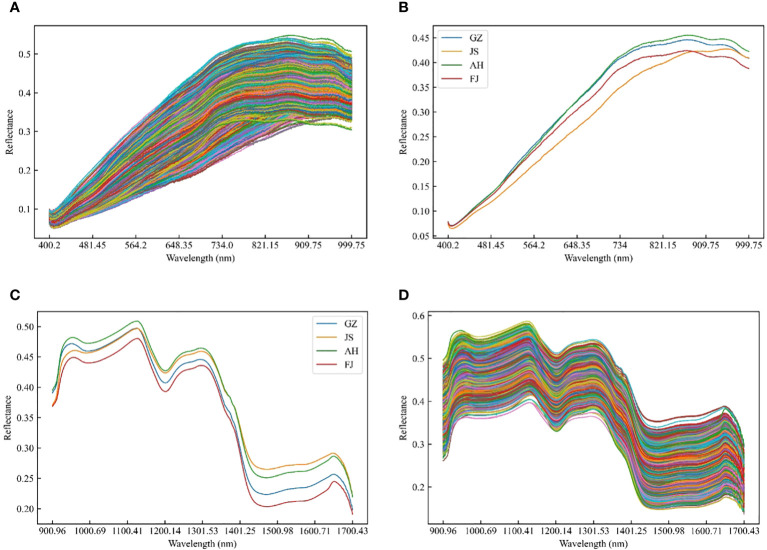
Raw and average spectra of TZS samples in the range of Vis/NIR and SWIR. **(A)** Raw spectra and **(C)** average spectra of TZS samples in the range of Vis/NIR; **(B)** Raw spectra and **(D)** average spectra of TZS samples in the range of SWIR.

The raw reflectance spectra of all the TZS samples from different origins were presented in **Figure 2**, covering the spectral ranges of 400-1000 nm and 1000-2000 nm. For the same spectral range, the TZS from different origins exhibited similar trends in general. This was similar to the previous researches conducted on discriminating maize varieties, determining the geographical origin of wolfberries, and assessing the quality of potatoes (López-Maestresalas et al., 2016; Dong et al., 2022). Although the spectral curves exhibited similar trends across various origins, there were variations in reflectance intensities. This discrepancy suggested that while the types of internal substances were similar, their concentrations differed among the different origins.

### 3.2 Preliminary principal component analysis to explore natural clustering of TZS samples

**Figure 3:**
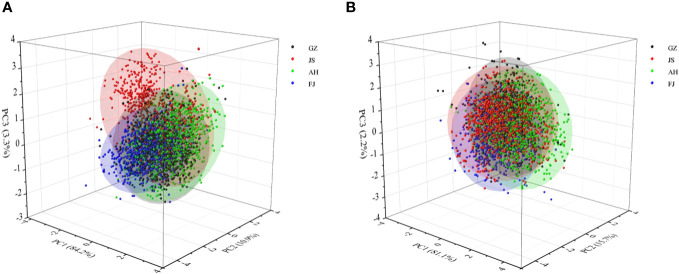
Scores scatter plots of Vis/NIR and SWIR spectra of TZS from four geographical origins. **(A)** Vis/NIR spectra; **(B)** SWIR spectra.

Two Principal Component Analysis (PCA) models were initially developed using the Vis/NIR and SWIR spectra of the TZS samples with the aim of observing the initial structure of the data from different geographically originated samples and detecting any anomalies among the samples. Three principal components (PCs) were selected for the Vis/NIR range, which accounted for 98.4% of the total variance (**Figure 3A**). Similarly, three PCs were chosen for the SWIR range, explaining 99.0% of the total variance (**Figure 3B**). According to the analysis, significant overlap between samples from different origins was observed in both spectral regions. It was worth noting that the distribution patterns of samples from different origins between the two spectral regions varied to some extent. In the Vis/NIR range, the samples of TZS from Jiangsu origin were slightly separated from the samples of other origins. Yet, this was not observed in the SWIR region. These differences provided a theoretical basis for further in-depth mining of the data in the two spectral regions separately.

### 3.3 Classification models based on full wavelengths

**Figure 4:**
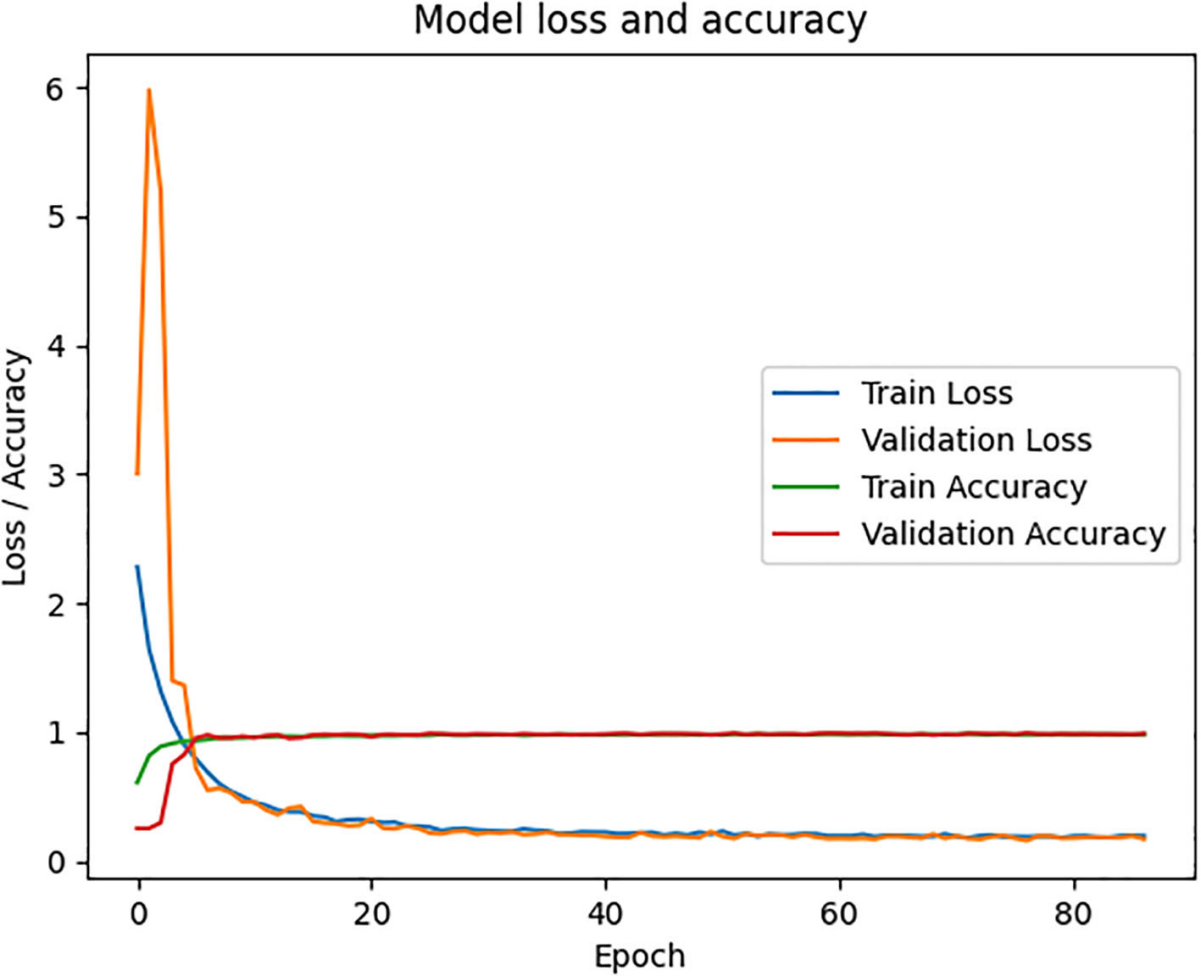
The loss and accuracy curves of the FD-DCNN model based on the SWIR.

**Figure 5:**
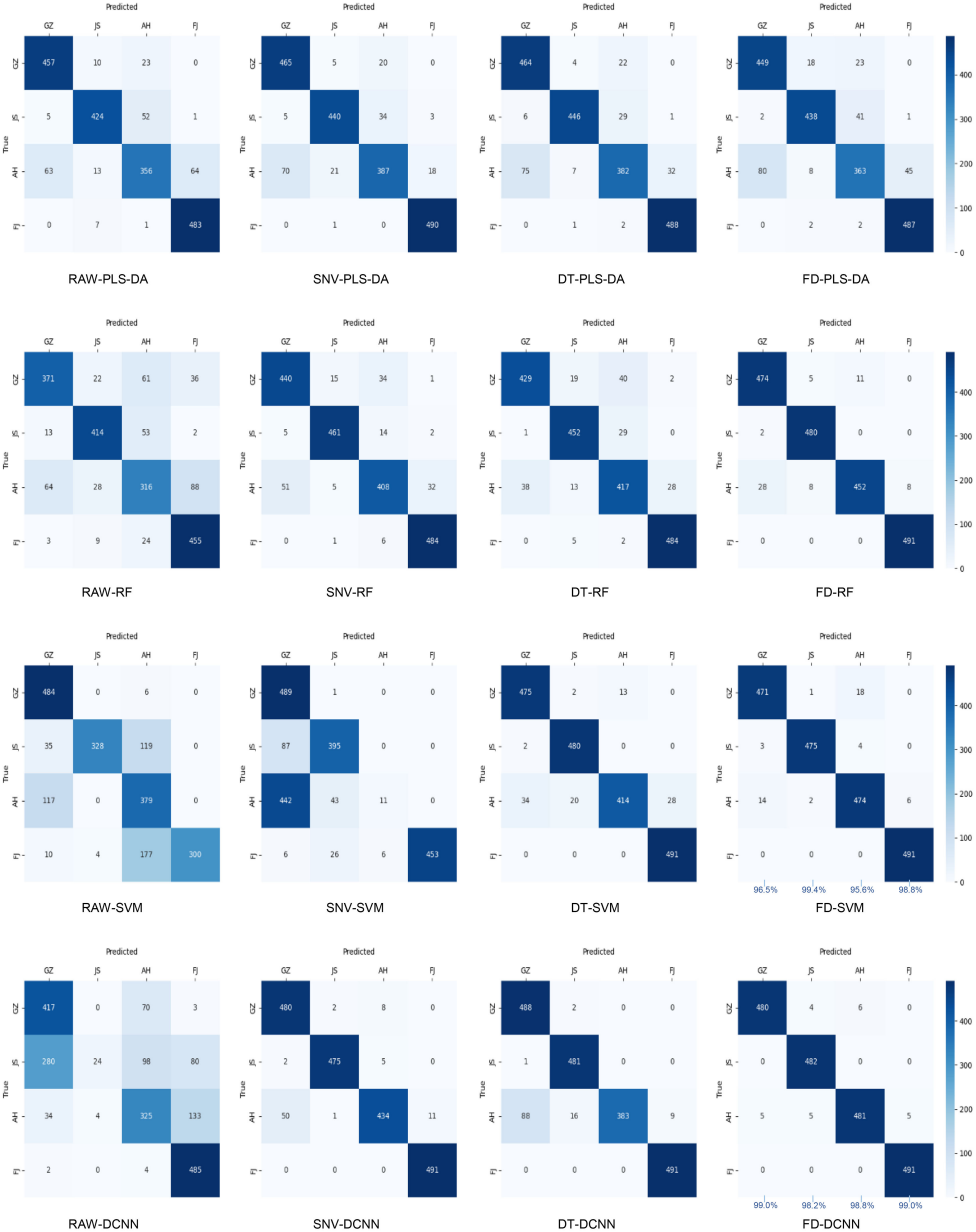
The confusion matrices of the PLS-DA, SVM, RF and DCNN models on the prediction set using different preprocessed SWIR spectra.

The PLS-DA, RF, SVM, and DCNN classification models were constructed by combining the SNV, DT and FD algorithms based on the spectral data from the Vis/NIR and SWIR regions as the input matrices, respectively (**Table 2**). The loss and accuracy curves for the optimal model were depicted in **Figure 4**. The loss value of the discriminative model continuously decreased as the number of iterations increased. Still, the precision increased and ultimately stabilized, indicating that the FD-DCNN model converged properly. In all cases, the discriminative performance of the models using SWIR spectra was superior to those using Vis/NIR spectra. The prediction accuracies of the models in the Vis/NIR region were lower than 91.4%, while the SWIR models could reach 98.7% accuracies. The selection of sensor type (information source), pretreatment methods, and classifier collectively influence the discrimination accuracy of the models to varying extents. Compared to the SNV and DT algorithms, the FD algorithm exhibited superior preprocessing performance in both the visible and near-infrared regions. This suggested that FD might be an ideal preprocessing method to improve the signal-to-noise ratio of spectra associated with the origin of TZS. Additionally, in the SWIR range, the model built with DCNN combined with FD pretreatment algorithm exhibited the highest precision, achieving 98.7% discrimination accuracy for the four origins of the TZS samples. Interestingly, the FD-SVM and FD-RF models also obtained satisfactory classification accuracies with validation sets of 97.6% and 96.8%, respectively. The confusion matrices of the models for the SWIR region were illustrated in **Figure 5**, which revealed that the FD-SVM and FD-DCNN models not only achieved desirable accuracies in terms of total correctness, but their accuracies were still high (>95.6%) for each origin category. With regard to the application of the model, we sought to reduce both the associated equipment costs and the time required for model prediction. Consequently, further research was carried out to extract a smaller number of feature bands from the SWIR spectra to establish a more efficient discrimination method.

**Table 2:** Results of classification models based on individual spectral region datasets.

Ranges	Models	Treatments	Parameters	Classification accuracy (%)	
				Training set	Validation set
Vis/NIR	PLS-DA	Raw	LV: 12	88.6	86.1
		SNV	LV: 9	84.3	82.9
		DT	LV: 6	66.6	66.7
		FD	LV: 10	88.3	85.7
	RF	Raw	T: 200; L: 1	100.0	71.1
		SNV	T: 200; L: 1	100.0	81.6
		DT	T: 200; L: 1	100.0	79.1
		FD	T: 200; L: 1	100.0	85.1
	SVM	Raw	C: 1000000.0, gamma: 0.0001	98.0	56.2
		SNV	C: 100000.0, gamma: 0.0001	98.1	80.5
		DT	C: 1000000.0, gamma: 1e^-05^	98.0	73.7
		FD	C: 100000.0, gamma: 1e^-06^	96.1	90.0
	DCNN	Raw	—	68.9	68.5
		SNV	—	64.7	63.4
		DT	—	70.5	69.4
		FD	—	94.8	91.4
SWIR	PLS-DA	Raw	LV: 7	86.6	87.8
		SNV	LV: 8	88.6	90.9
		DT	LV: 8	88.7	90.8
		FD	LV: 8	88.2	88.7
	RF	Raw	T: 200; L: 1	100.0	79.4
		SNV	T: 200; L: 1	100.0	91.5
		DT	T: 200; L: 1	100.0	90.9
		FD	T: 200; L: 1	100.0	96.8
	SVM	Raw	C: 464158.8, gamma: 1e^-05^	97.1	76.1
		SNV	C: 100000.0, gamma: 0.0001	98.9	68.8
		DT	C: 10000000.0, gamma: 1e^-06^	98.3	95.0
		FD	C: 10000.0, gamma: 1e^-05^	98.3	97.6
	DCNN	Raw	—	68.3	67.6
		SNV	—	93.0	93.4
		DT	—	94.9	94.1
		FD	—	99.6	98.7

### 3.4 Selection of effective wavelengths

An appropriate wavelength selection method is crucial as it not only reduces the number of wavebands but also helps eliminate irrelevant, noisy, or collinear variables, thereby improving the modeling precision (Liu et al., 2022). Moreover, different wavelength selection methods are based on different algorithm principles, which can lead to varying modeling results when applied to different types of datasets. It is important to carefully consider the characteristics of the dataset and choose a wavelength selection method that best suits the specific needs. In this study, CARS and SPA were utilized to select the effective wavelengths (EWs) from the SWIR spectra that would potentially contain the most valuable information regarding the geographical origins of TZS samples.

**Figure 6:**
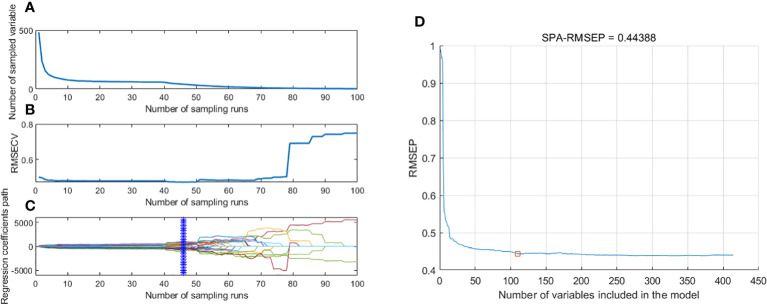
The process of extracting EWs with CARS and SPA. **(A)** Number of preferred EWs with CARS; **(B)** The root mean square error of cross-validation variation with CARS; **(C)** Regression coefficient path map with CARS; **(D)** Extraction of EWs with SPA.

The randomness of the Monte Carlo sampling resulted in inconsistent results for every operation of the CARS approach and the optimal results after 10 CARS selections were chosen to obtain a relatively optimal combination of bands (**Figure 6**). Under the exponential decay function, the number of bands decreases rapidly at the beginning of the sampling, but with the sampling number increasing, the rate of decrease of the band number slows down gradually (**Figure 6A**). As shown in **Figure 6B**, the RMSECV values showed an overall trend of decreasing and then increasing with the sampling times, and the RMSECV values were the lowest when the number of sampling times reached 47. Combined with **Figure 6C**, it was observed that the RMSECV value was the smallest at the 47th sampling (* denotes), meaning that the subset containing 32 variables selected for this sampling was the key to determining the origins of TZS. The SPA method establishes a multiple linear regression model for different subsets of bands one by one and calculates the RMSEP values when selecting the optimal bands, where the subset corresponding to the smallest value is the optimal subset of bands. As shown in **Figure 6D**, the RMSEP values show an overall decreasing trend with the increase in the number of bands. When the number of bands reaches 110, the RMSEP value minimizes to 0.444 and then slightly increases. The specific descriptions of the EWs screened with the CARS and SPA algorithms are listed in **Table 3**.

**Table 3:** Specific description of the selected EWs by SPA and CARS.

Methods	No.	EWs							
CARS	32	919.24	930.88	934.2	935.86	959.13	969.11	975.75	1117.03
		1130.33	1131.99	1143.63	1148.61	1150.28	1168.56	1175.21	1245.02
		1286.57	1341.42	1377.98	1381.31	1382.97	1384.63	1391.28	1407.9
		1409.56	1417.88	1421.2	1434.5	1574.11	1632.29	1635.61	1640.6
SPA	110	907.61	909.27	910.93	922.57	924.23	929.21	934.2	940.85
		945.84	947.5	949.16	954.15	955.81	959.13	965.78	969.11
		987.39	989.05	990.71	1002.35	1004.01	1005.67	1007.33	1012.32
		1017.31	1018.97	1020.63	1025.62	1027.28	1032.27	1033.93	1035.59
		1043.9	1048.89	1050.55	1058.86	1062.18	1063.85	1065.51	1078.8
		1080.47	1092.1	1093.76	1095.43	1105.4	1108.72	1110.38	1113.71
		1115.37	1118.7	1120.36	1128.67	1131.99	1138.64	1145.29	1151.94
		1158.59	1188.5	1213.44	1215.1	1218.42	1228.39	1230.06	1240.03
		1241.69	1245.02	1256.65	1283.24	1284.91	1311.5	1313.16	1328.12
		1329.78	1333.11	1341.42	1346.4	1349.73	1361.36	1364.69	1366.35
		1369.67	1376.32	1377.98	1384.63	1391.28	1407.9	1414.55	1419.54
		1431.17	1432.83	1436.16	1437.82	1446.13	1487.68	1489.35	1491.01
		1492.67	1495.99	1502.64	1504.3	1505.97	1509.29	1510.95	1584.09
		1600.71	1628.96	1638.94	1643.92	1645.58	1648.91		

### 3.5 Classification models based on EWs

**Figure 7:**
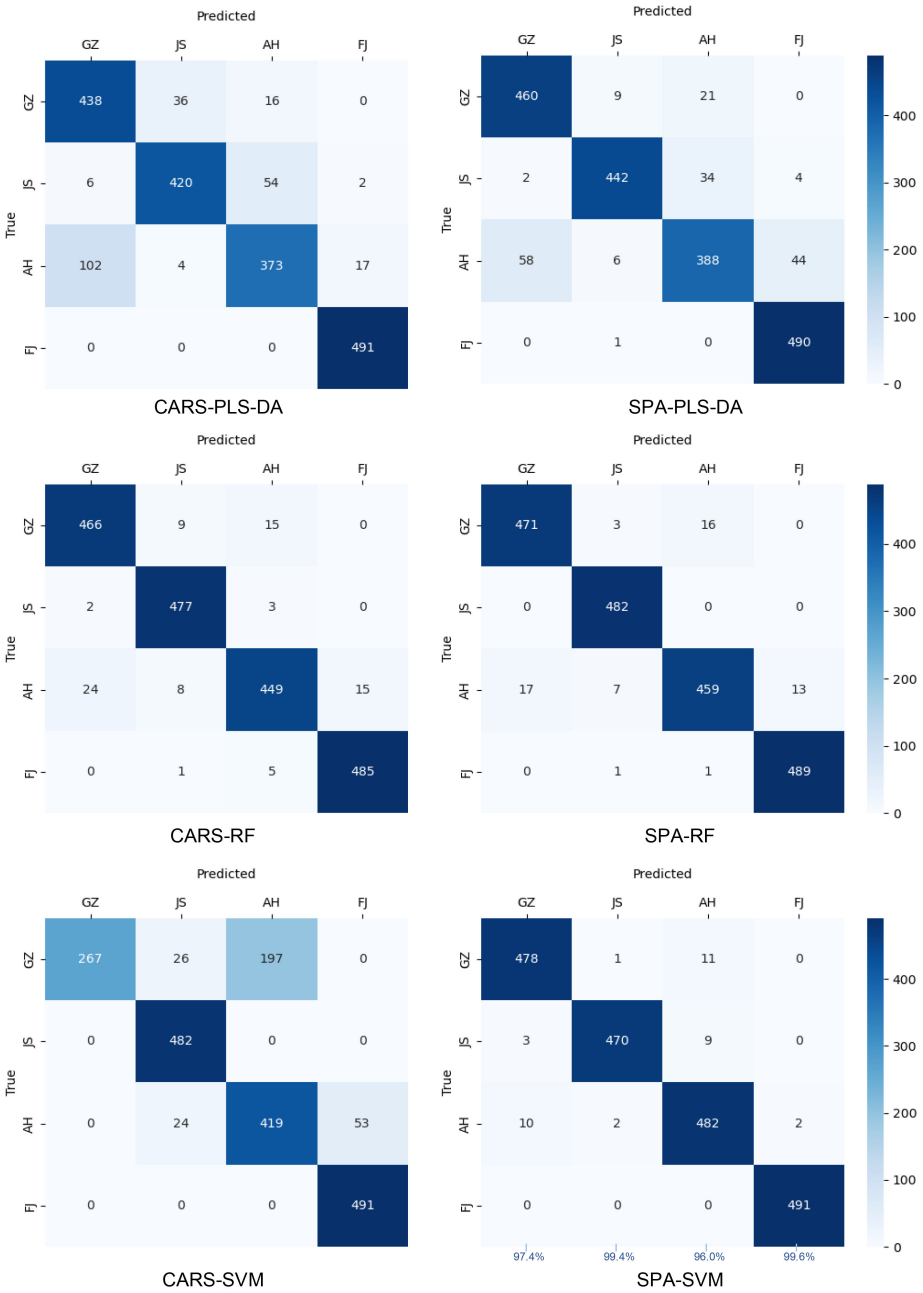
The confusion matrices of the simplified PLS-DA, SVM and RF models on the prediction set.

After applying the CARS and SPA algorithms to select the essential wavelengths (EWs), simplified PLS-DA, RF and SVM models were developed to determine the geographic origins of TZS (**Table 4** and **Figure 7**). The models exhibited different performances, indicating that the choice of wavelength selection method had varying effects on the discriminative models of TZS origins. The performance of the CARS-RF and SPA-RF models exhibited a slight degradation compared to the full-band FD-RF model. Additionally, both SVM and RF models based on the CARS method performed worse than the SPA-based SVM and RF models. This discrepancy might be attributed to the limited number of EWs selected by CARS, leading to the elimination of some EWs containing crucial information about the TZS origins. Remarkably, the SPA-SVM model based on 110 EWs obtained the optimal discriminative accuracy with 98.4% for the training set and 98.1% for the validation set. Although it was slightly worse than the full-band FD-DCNN model (training set of 99.6% and validation set of 98.7%), it outperformed the full-band FD-SVM model (training set of 98.3% and validation set of 97.6%). These results indicated that compared to CARS, the SPA algorithm is preferable for extracting the SWIR information that is highly correlated with the TZS origins.

**Table 4:** Results of simplified classification models based on SWIR spectra.

Models	EWs selection methods	Number of EWs	Parameters	Classification accuracy (%)	
				Training set	Validation set
PLS-DA	CARS	32	LV: 7	86.1	87.9
	SPA	110	LV: 9	89.2	90.9
RF	CARS	32	T: 50; L: 1	100.0	95.8
	SPA	110	T: 50; L: 1	99.9	97.0
SVM	CARS	32	C:10000000.0, gamma: 1e^-06^	97.6	84.7
	SPA	110	C:10000.0, gamma: 0.0001	98.4	98.1

An additional analysis was performed on the extracted EWs from SPA, as shown in **Table 3**. This analysis revealed that most of the EWs were concentrated in specific regions of the spectra, indicating a potential relationship between the origin and chemical composition of TZS. The wavebands around 970 nm are associated with O-H second overtone stretching vibration and C-H stretching third overtone, which are related to sugar and cellulose, respectively (Theanjumpol et al., 2013). The bands between 1050 nm and 1200 nm, as well as 1300 nm and 1500 nm, are the main characteristic spectral regions that represent the 20 amino acids found in proteins. The 1050-1200 nm region primarily consists of the second overtone of C-H, while the 1300-1500 nm region is mainly composed of the combined frequency of C-H, reflecting the differences in amino acid composition among different samples (Weinstock et al., 2006; Jin et al., 2022).

### 3.6 Optimal model validation and visualization

**Figure 8:**
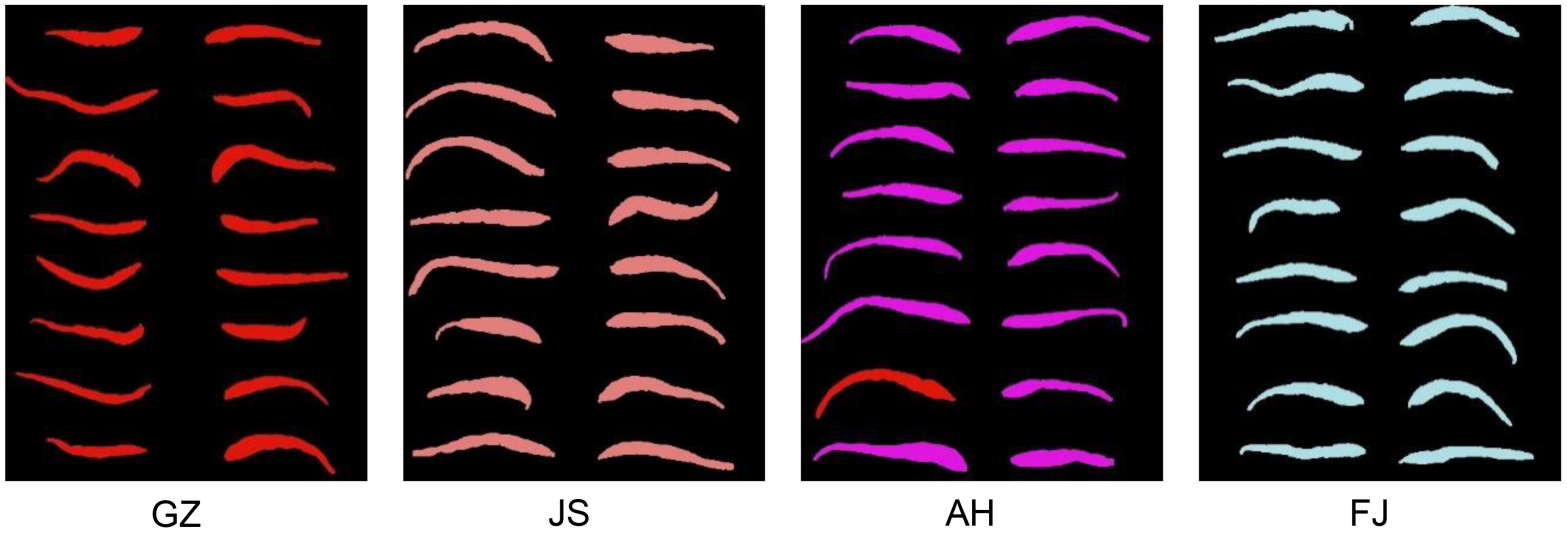
Detection visualization of TZS samples from Guizhou (GZ), Jiangsu (JS), Anhui (AH) and Fujian (FJ) Provinces.

Apart from the 3249 TZS samples used for modeling, an additional 320 samples (80 TZS per origin) were selected for external validation and visualization of the optimal model (FD-DCNN). The visualization of the validation results is shown in **Figure 8**. The origin of TZS was marked with different colors, with red representing TZS identified by the model as originating from Guizhou (GZ) Province, pink representing TZS identified by the model as originating from Jiangsu (JS) Province, purple representing TZS identified as originating from Anhui (AH) Province, and blue indicating TZS identified as originating from Fujian (FJ) Province. It can be seen that all TZS from Guizhou, Jiangsu, and Fujian provinces were correctly recognized (100%). One sample in the Anhui group was incorrectly identified as TZS from Guizhou Province with a precision of 98.8%. The results of this external validation were consistent with the results of the FD-DCNN model, indicating that the discrimination model developed in this study for TZS had excellent robustness.

Previous studies on the discrimination of the origin of TZS are based on only one variety from one origin (Wu et al., 2018; Pan et al., 2020), overlooking the disturbances caused by the genetic background and grade differences, which leads to the limited application of the model. Our methodology considered the representativeness of the samples and the applicability of the approach by selecting two or three germplasm resources for each geographical region. Furthermore, each germplasm resource included different quality grades of TZS (**Table 1**), enhancing the comprehensiveness of the analysis.

In the analysis of the two spectral ranges used in this study, the models within the range of 900.96~1700.43 nm demonstrated superior performance compared to the models within the 400.20~999.75 nm range. The correct classification rates for the prediction set ranged from 67.6% to 98.1% for the 900.96~1700.43 nm range (**Tables 2**, **4**), while they ranged from 56.2% to 91.4% for the 400.20~999.75 nm range (**Table 2**). This difference in accuracy can be attributed to the fact that the spectra in the 900.96~1700.43 nm range provide information about the stretching vibrations of C-H, O-H, and N-H, which are caused by starch, protein, cellulose, and water in the TZS. On the other hand, the wavelengths between 400.20 and 999.75 nm primarily reflect the color and pigment information in the TZS. Hou et al. (2015) conducted an analysis of the chemical compositions in P. *heterophylla* from different origins using UPLC-Triple TOF-MS/MS. The study identified 21 distinct chemical components, including maltotriose, sucrose, thyronine, inosine triphosphate, pseudostellarin A, pseudostellarin B, pseudostellarin D, pseudostellarin F, heterophyllin A and sphinganine. Compared with other origins, the levels of pseudostellarin D, pseudostellarin E, pesudostellarin A, heterophyllin A, pseudostellarin F, isobutyrylglycine in P. *heterophylla* from Fujian were higher. Sucrose, ferulic acid, canthaxanthin, maltotriose, pseudostellarin D in P. *heterophylla* from Guizhou were richer than those of other origins (Sha et al., 2023). Hence, it is reasonable to hypothesize that spectral differences resulting from variations in chemical composition, rather than color and pigmentation information, may play a crucial role in studying the traceability of the origin of *Pseudostellaria heterophylla*.

Notably, our work takes a novel approach by using hyperspectral imaging (HSI) in conjunction with deep learning (DL) techniques to assess the geographical origins of TZS. Wu et al. (2018) highlighted the efficiency of using Raman spectroscopy combined with MSC-SG-CARS-PLS-DA to discriminate P. *heterophylla* from different regions. Similarly, Pan et al. (2020) demonstrated that NIR spectroscopy in combination with Row-center-SG-CARS-PLS-DA could be effective in distinguishing the P. *heterophylla* from different regions. Further to this, this research conducted a comparison between two feature band extraction algorithms, namely CARS and SPA. The results showed that the SPA algorithm was preferable for extracting SWIR information, which was highly correlated with the TZS origins (**Table 3** and **Table 4**). Furthermore, we compared the traditional two-stage machine learning algorithms (PLS-DA, SVM, and RF) with the end-to-end deep learning algorithm (DCNN). Our findings demonstrated that both SVM and DCNN classifiers outperformed PLS-DA and RF classifiers in terms of origin identification of TZS (**Table 2** and **Figure 5**). Several previous studies indicated that nonlinear models, such as SVM, were superior to linear models in solving the seed classifications (Qiu et al., 2018; Wakholi et al., 2018; Zhao et al., 2018). For the first time, our work further argued this on geographic origin recognition in TZS.

## 4 Conclusion

In this study, the visible near-infrared (Vis/NIR) and short-wave infrared (SWIR) hyperspectral information from different origins of TZS samples were collected. By combining various preprocessing algorithms, feature band extraction algorithms, traditional two-stage machine learning, and end-to-end deep learning classifiers, we developed fast and high-precision identification methods to discriminate TZS origins. The specific conclusions drawn from this study are as follows:

1) The model accuracy based on SWIR HSI for identifying the geographical origins of TZS was higher compared to that based on Vis/NIR HSI. The best model accuracy using Vis/NIR HSI was 91.4%, while the optimal model accuracy using SWIR HSI could reach up to 98.7%.
2) The SPA algorithm was suitable for extracting SWIR information, which was highly correlated with the origins of TZS. The corresponding FD-SPA-SVM model not only reduced the number of bands by 77.2% but also improved the model accuracy from 97.6% to 98.1% compared to the full-band FD-SVM model.
3) Two sets of fast and high-precision methods were developed to distinguish between different geographic origins of TZS. The traditional two-stage machine learning classifier achieves optimal performance by employing the SVM model with FD pretreatment and the variable selection method of SPA. In contrast, the end-to-end deep learning classifier achieves optimal discrimination by solely applying FD preprocessing combined with DCNN. The total accuracies of the SWIR-FD-SPA-SVM model and the SWIR-FD-DCNN model for identifying TZS origins were 98.1% and 98.7%, respectively.

This work provides a potentially perfect tool for herbal companies and market regulators to widely identify the origins of TZS across various genetic backgrounds and quality grades.

## Data availability statement

The original contributions presented in the study are included in the article/supplementary material. Further inquiries can be directed to the corresponding authors.

## Author contributions

TZ: Conceptualization, Methodology, Supervision, Visualization, Writing – original draft, Writing – review & editing. LL: Data curation, Formal analysis, Writing – review & editing. YS: Data curation, Writing – original draft. MY: Writing – review & editing. JL: Writing – review & editing. JY: Writing – review & editing. YL: Writing – review & editing. XS: Writing – review & editing. ML: Writing – review & editing. XY: Writing – review & editing. ZZ: Writing – review & editing. RZ: Writing – review & editing. YuS: Funding acquisition, Supervision, Writing – review & editing. LG: Funding acquisition, Supervision, Writing – review & editing.
